# Abdominal Aortic Neck Wrap for Refractory Type 1a Endoleak: A Case Series and a Novel Intraoperative Assessment Technique

**DOI:** 10.3400/avd.oa.20-00152

**Published:** 2021-03-25

**Authors:** Ali Kordzadeh, Tamer Sayed, Manfred J. Ramirez, Ioannis Prionidis, Adam Howard, Tom Browne

**Affiliations:** 1 Department of Vascular and Endovascular Surgery, Broomfield Hospital, Chelmsford, CM1 7ET, United Kingdom; 2 East Suffolk & North Essex NHS Foundation Trust, Colchester General Hospital, Tumer Road, Colchester, Essex, C04 5JL, United Kingdom

**Keywords:** refractory type Ia endoleak, wrap aortic neck, banding, novel assessment technique, Doppler waveform

## Abstract

**Objective**: Refractory type 1a endoleak after endovascular aneurysm repair (EVAR) can pose a significant challenge to surgeons and interventional radiologists. Continuous sac expansion results in aneurysm rupture and mortality. In such circumstances, an external infrarenal aortic wrap could serve as an essential and alternative solution.

**Methods**: We assessed the application of an infrarenal aortic neck wrap for the treatment of refractory type 1a endoleak in n=6 consecutive patients along with the introduction of a novel assessment technique in order to assure their intraoperative success with no radiation exposure and contrast use.

**Results:** The median sac expansion was 8.5 mm (interquartile range [IQR], 5–20 mm). The median neck diameter and length of the aortic neck were 23 mm (IQR, 18–25 mm) and 21 mm (IQR, 18–25 mm), respectively. The median length of follow-up post wrap is 24 months (IQR, 14–34 months). There was no associated mortality or morbidity and requirement for any further interventions.

**Conclusion**: The study demonstrates that aortic wrapping for the treatment of refractory type 1a endoleak for any given neck diameter and length is safe, effective, and long lasting. The suggested novel intraoperative assessment technique contributes to the safety of the procedure by diminishing the need for intraoperative radiation exposure, contrast, and shorter operative time.

## Introduction

Endovascular aneurysm repair (EVAR) is now an accepted and widespread modality for the repair of infrarenal abdominal aortic aneurysms (AAAs). Endoleak is a well-known complication of EVAR and occurs in an estimated 15%–52% of all EVAR cases. Type 1a endoleak is uncommon (1%–3%), and it is attributed to an incomplete seal of endograft, difficult neck anatomy, and aneurysmal degeneration. Type 1a endoleak is associated with a high risk of sac expansion and rupture.^1–4)^ Once further endovascular options fail to prevail, open surgery remains the only option and in some centers, endograft explantation might be attempted, which is associated with significant mortality and morbidity.^1–4)^ Herein, we would like to report six (n=6) successful cases of external wrap and/or banding of the infrarenal aortic neck for refractory type 1a endoleak with a novel intraoperative assessment technique to assure their success.

## Methods

A prospective data collection on six individuals (n=6) that required external wrapping/banding for type 1a endoleak from January 2012 to March 2019 was performed. All cases had sac expansions due to delayed and refractory type 1a endoleak. Data on patient’s demographic, AAA size, sac expansion, comorbidities, endograft type, the American Society of Anesthesiology (ASA) score, blood loss, length of operation (LOP), length of stay, outcome, and follow-up were recorded. In our department, all patients following EVAR are subjected to a fixed follow-up protocol for 9 years. Initially, all patients are followed up via computed tomography angiography (CTA), and once endoleak is ruled out, duplex sonography remains the investigative modality of the choice. Upon detection of endoleak (types I–IV), CTA is accompanied by contrast enhanced ultrasound (CEUS) to monitor possible sac expansion and/or their resolution. Sac growth definition (at least 5 mm) was according to recommended SVS reporting standards.^5)^ The presence of type 1a Endoleak was confirmed in a multidisciplinary team (an interventional radiologist and a vascular surgeon) by the evaluation of the CTA and CEUS images with consensus on the sac expansion and other respective measurements. The current rate of type I endoleak in our department for 12 years is 4.1% of n=250 EVAR cases (adherent to instruction for use) with time to event (type 1a) of 7 years (interquartile range [IQR], 1–12 years), and there appears to be a direct relationship between the neck angulation of >60°, aneurysmal sac changes, and use of anticoagulation to that of type 1a occurrence. This study was approved by the local research and audit committee along with accompanying images (CA19-024) without enclosing patients identifying factors. The study was conducted following the Helsinki code of ethical principles, and no patient was subjected to any new, alternative, or change of practice.

### Surgical technique

**Figure 1:**
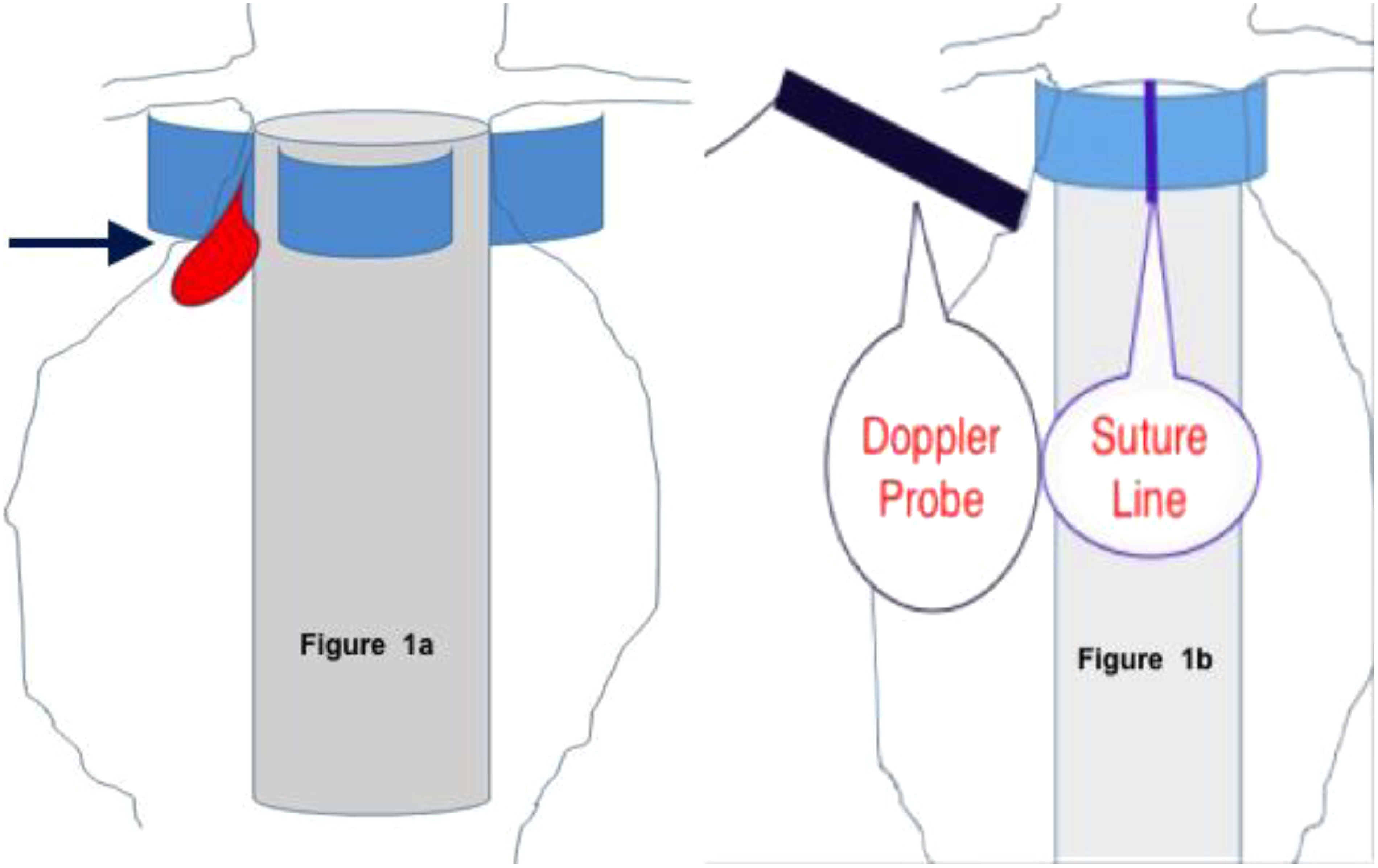
Fig. 1 (**a**) Demonstration of the aneurysm sac with Endoleak Type 1a (arrow) along with the Dacron wrap in position for suturing anteriorly. (**b**) Demonstration of how the Doppler probe is applied before tightening the wrap over the aorta. The probe is placed at an angle of >30° but less than <90°. The characteristic of the waveform Doppler is almost unidirectional high-peak turbulent flow and/or jet flow, and if limited to type 1a endoleak, it is not to–fro (yin yang sign).

All six patients (n=6) had general anesthesia and a mini upper laparotomy. After careful dissection and identification of the renal vessels (left renal vein and arteries), the aorta is circumferentially dissected for 5–7 cm longitudinally from its infrarenal aortic neck. The reason behind this is threefold: First, it permits adequate control circumferentially (in case of rupture or tear); second, it avoids slippage and bottleneck effect of wrap (fold back of the wrap on its own inferiorly) or pressurizing renal vessels superiorly; and third, it allows direct visualization for any lumbers that were not detected in preoperative CTA. In case of the identification of any lumber arteries, they could be clipped, ligated, or transfixed. However, in our series, we did not encounter any. A Dacron (MAQUET, HEMASHIELD PLATINUM, Woven Double Velour Grafts, NJ, USA) graft is then measured and cut into appropriate length and width and passed under the aorta with both ends brought anteriorly. This is secured in position using polypropylene monofilament suture 2/0 (PROLENE™) over another pre-cut sheet of Dacron graft (1 cm wide and 5–7 cm long) (double breasting) to the adventitia of the aorta (**Fig. 1**). This avoids direct pressure over the aorta from the stitch line, inhibits tear, and re-enforces the wrap in position. The retro-peritoneum is then closed over the graft, and the omentum is placed between the duodenum and the wrap to avoid erosion. The procedure in all six individuals was performed without aortic cross-clamping, and the abdomen was closed in a standard fashion.

### Novel assessment technique to evaluate the success

According to the reported series in the literature, the success of the intervention (the external wrapping/banding of the aorta in type 1a endoleak) is evaluated by subjecting individuals to intraoperative angiography and/or CTA in a hybrid setting before closure.^6)^ In some series, repeated angiographies had to be performed to evaluate the success of the wrap.^7)^ This resulted in an additional procedure for access and wiring with further exposure (patient and surgeon) to radiation and the use of contrast.

In our department, we assess the success of the wrap by intraoperative handheld Doppler assessment. In this technique, the Doppler probe is placed on the aneurysm sac proximally. This detects active waveforms from the endoleak 1a (in the absence of any other endoleak). The probe is placed at an angle of >30° but less than <90°. The characteristic of the waveform Doppler is almost unidirectional high-peak turbulent flow and/or jet flow, and if limited to type 1a endoleak, it is not to–fro (yin yang sign). Once the wrap is in position, it is tightened until the waveform disappears. At this stage, the wrap is secured in position with sutures. This step was also initially evaluated via intraoperative Doppler sonography. This novel and simple assessment technique diminishes the need for intraoperative angiography and/or CTA, omits radiation exposure, avoids the use of contrast, shortens the procedure time, and can be performed in non-hybrid operating theaters (**Fig. 1**).

### Statistical analysis

All statistical analysis was conducted using the statistical package for the social sciences version 20, IBM. All continuous variables were reported as median with their corresponding IQRs and percentages. The outcome of each procedure and their respective follow-up period was reported in the median with their IQRs.

## Results

The majority of patients were male (n=5/6, 83%) with a median age of 77 years (IQR, 74–81 years). The most common comorbidity was hypertension (n=6, 100%) followed by ischemic heart disease (n=4, 67%) and chronic obstructive pulmonary disease (n=4, 67%). One patient suffered from rate-controlled atrial fibrillation (n=1, 16%) and another from congestive heart disease (n=1, 16%). The ASA score was 3.

**Figure 2:**
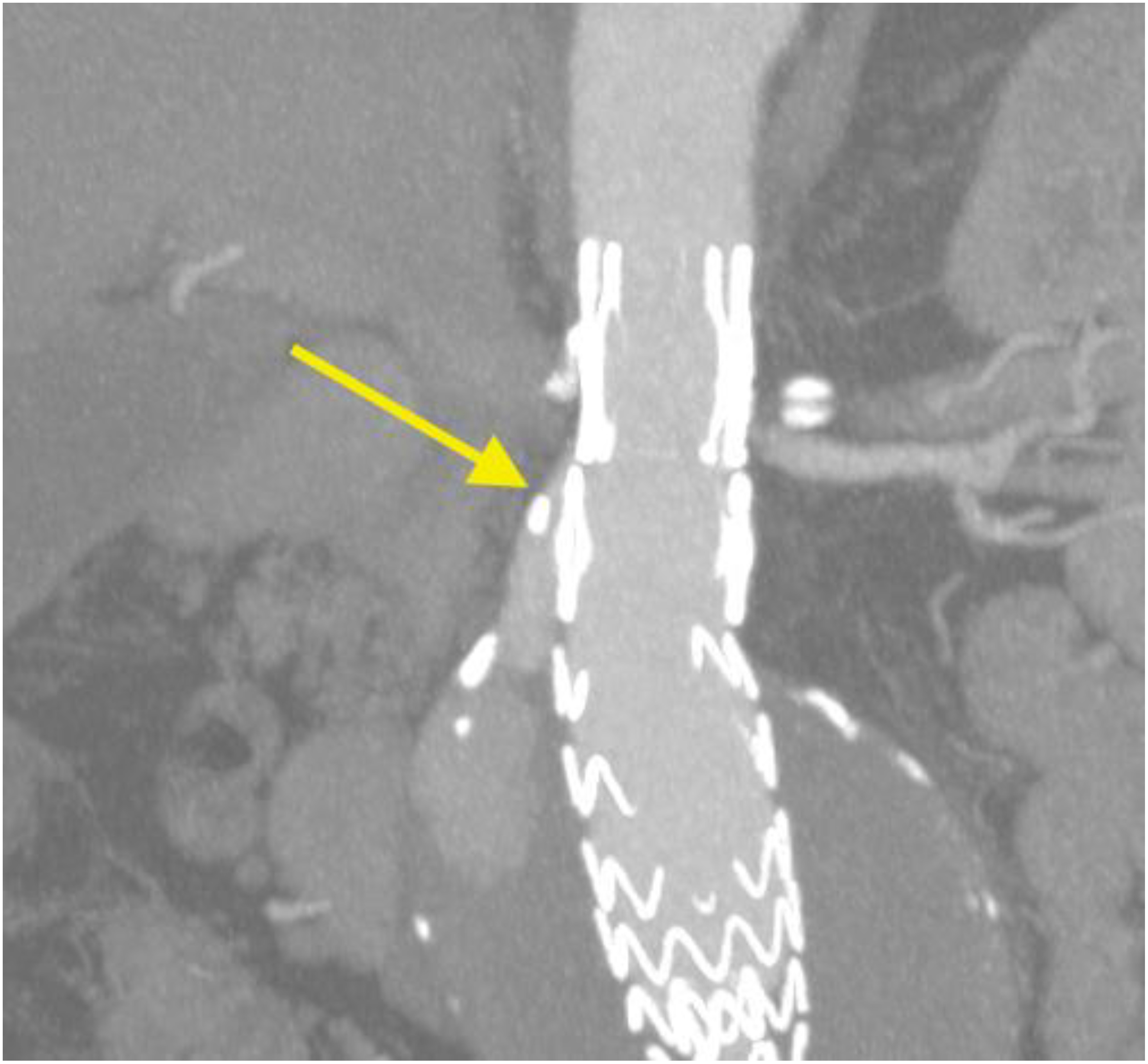
Fig. 2 Preoperative computed tomography angiography of active refractory type 1a endoleak.

**Figure 3:**
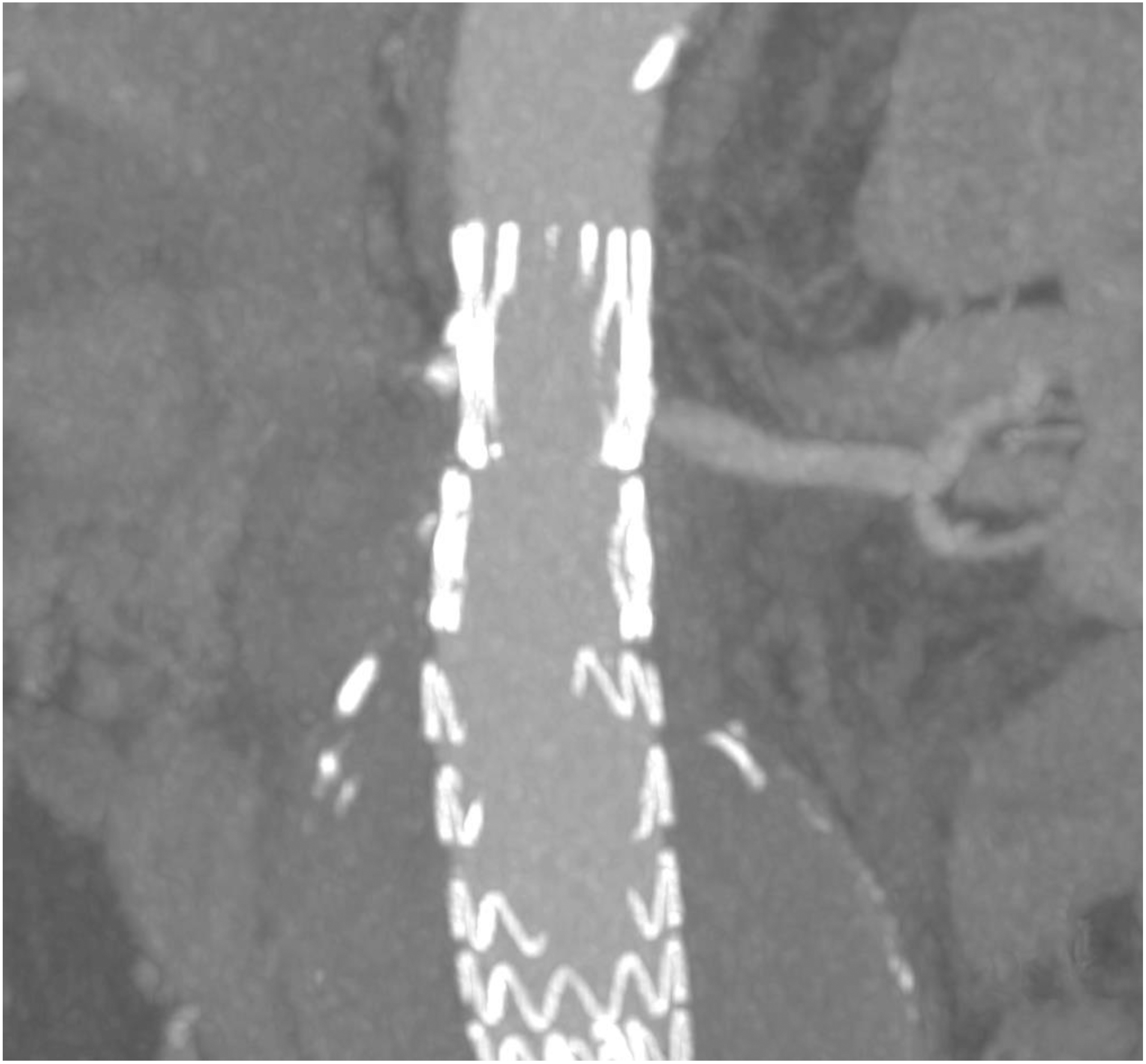
Fig. 3 Postoperative computed tomography angiography image demonstrating the resolution of the refractory type 1a endoleak following wrap technique.

The median sac expansion was 8.5 mm (IQR, 5–20 mm) from the original aneurysm size (before EVAR) of 60 mm (IQR, 55–66 mm). The median diameter of the aortic neck was 23 mm (IQR, 18–25 mm) and this value for neck length was 21 mm (IQR, 18–24 mm) (**Fig. 2**). The median intraoperative blood loss was 275 ml (IQR, 100–350 ml), and the LOP was 87.5 min (IQR, 80–100 min). The length of in-hospital stay was 5 days (IQR, 3–6 days). There was no postoperative mortality and/or morbidity. The median length of follow-up without endoleak type 1a post wrap has been 24 months. The overall median postoperative sac shrinkage was 3.5 mm (IQR, 0–5 mm) with no sac expansion. No patient required any additional procedures following the wrap (**Table 1**) (**Fig. 3**). However, one patient died from metastatic squamous cell carcinoma of the skin 14 months after the procedure.

**Table 1:** Table 1 Details of each patient with type Ia refractory endoleak

Patient	Sex (male/female)	Age (years)	Length of stay (days)	Neck length (mm)	Neck diameter (mm)	Aneurysm size (mm) before EVAR	Sac expansion (mm) Pre	Sac reduction (mm) Post
1	M	81	3	25	25	65	20	3
2	M	75	5	24	25	66	5	5
3	M	81	5	18	21	56	8	5
4	M	75	5	21	22	55	9	0
5	M	78	6	18	23	60	10	0
6	F	74	5	21	18	60	8	4

## Discussion

In our unit, all type 1a endoleaks with sac expansion are initially evaluated for the placement of aortic extension with or without suprarenal fixation. The endograft/stent of choice is Palmaz XL stent (Cordis endovascular, a Johnson and Johnson company, Piscataway, NJ, USA) for the aortic neck. Once they fail to achieve the desired (seal of endoleak) outcome or to be used (short necks), banding is therefore considered.

This case series demonstrates that external wrap of the infrarenal aorta following EVAR is an effective technique for the resolution of type 1a endoleak and can be equally achieved at any desired diameter and length. This technique was first introduced by Varcoe et al in 2008.^8)^ The approximate calculation before surgery can also be obtained using L=2πR formula on review of CTA images. The “L” represents the length of the graft and “2R” equates to the required diameter of the infrarenal aortic neck.

Contrary to other open modalities, this technique is far safer and maintains significant advantages. This is reflected in the lack of cross-clamping (short neck suprarenal and supraceliac), lack of ischemic time or long operative period, and, in worse-case scenarios, EVAR explantation.^9,10)^ Furthermore, the introduction of the new novel intraoperative assessment technique can maximize the benefits of the procedure by diminishing radiation exposure and/or contrast use.

Wrap could be also performed via the retro-peritoneum approach (laparoscopic or open surgery) reducing the extent of peritoneal dissection in complex abdomens with a minimal learning curve. However, the manipulation of the infrarenal aorta due to shaggy necks could result in a thrombus or atherosclerotic plaque dislodgement and, in some rare circumstances, the compression of the renal vessels due to slippage. Thus, with minimum manipulation and precise preoperative sizing, meticulous dissection around the aortic neck is highly advocated. Such technique could also be conducted via the laparoscopic approach, which could enhance patient recovery with shorter hospital stay. Overall, over a 24 month (median) period of follow-up, no patient exhibited any recurrence of type 1a endoleak in this series.

## Conclusion

The outcome of this series complements prior series on the efficacy, feasibility, and safety of aortic wrap/banding in the resolution of refractory type 1a endoleaks for any given diameter and length. The suggested novel intraoperative assessment technique contributes to the safety of the procedure by diminishing the need for intraoperative radiation exposure, contrast, and operative time.
